# Safety and efficacy of thrombectomy in patients undergoing primary percutaneous coronary intervention for Acute ST elevation MI: A Meta-Analysis of Randomized Controlled Trials

**DOI:** 10.1186/1471-2261-10-10

**Published:** 2010-02-26

**Authors:** Umesh U Tamhane, Stanley Chetcuti, Irfan Hameed, P Michael Grossman, Mauro Moscucci, Hitinder S Gurm

**Affiliations:** 1 Division of Cardiovascular Medicine, University of Michigan, Ann Arbor, MI, USA; 2 VA Ann Arbor Health Care System, Ann Arbor, MI, USA

## Abstract

**Background:**

Clinical trials comparing thrombectomy devices with conventional percutaneous coronary interventions (PCI) in patients with acute ST elevation myocardial infarction (STEMI) have produced conflicting results. The objective of our study was to systematically evaluate currently available data comparing thrombectomy followed by PCI with conventional PCI alone in patients with acute STEMI.

**Methods:**

Seventeen randomized trials (n = 3,909 patients) of thrombectomy versus PCI were included in this meta-analysis. We calculated the summary odds ratios for mortality, stroke, post procedural myocardial blush grade (MBG), thrombolysis in myocardial infarction (TIMI) grade flow, and post procedural ST segment resolution (STR) using random-effects and fixed-effects models.

**Results:**

There was no difference in risk of 30-day mortality (44/1914 vs. 50/1907, OR 0.84, 95% CI 0.54-1.29, P = 0.42) among patients randomized to thrombectomy, compared with conventional PCI. Thrombectomy was associated with a significantly greater likelihood of TIMI 3 flow (1616/1826 vs. 1533/1806, OR 1.41, P = 0.007), MBG 3 (730/1526 vs. 486/1513, OR 2.42, P < 0.001), STR (923/1500 vs. 715/1494, OR 2.30, P < 0.001), and with a higher risk of stroke (14/1403 vs. 3/1413, OR 2.88, 95% CI 1.06-7.85, P = 0.04). Outcomes differed significantly between different device classes with a trend towards lower mortality with manual aspiration thrombectomy (MAT) (21/949 vs.36/953, OR 0.59, 95% CI 0.35-1.01, P = 0.05), whereas mechanical devices showed a trend towards higher mortality (20/416 vs.10/418, OR 2.07, 95% CI 0.95-4.48, P = 0.07).

**Conclusions:**

Thrombectomy devices appear to improve markers of myocardial perfusion in patients undergoing primary PCI, with no difference in overall 30-day mortality but an increased likelihood of stroke. The clinical benefits of thrombectomy appear to be influenced by the device type with a trend towards survival benefit with MAT and worsening outcome with mechanical devices.

## Background

Primary percutaneous intervention (PCI) is the preferred reperfusion modality in patients with ST-elevation myocardial infarction (STEMI) [1]. While primary PCI is highly effective in achieving epicardial coronary reperfusion, a significant proportion of patients fail to achieve adequate myocardial reperfusion [2]. Measures of failed epicardial reperfusion such as persistent ST elevation or diminished myocardial blush grade (MBG) have demonstrated consistent association with poor left ventricular salvage and increased mortality and morbidity [3,4]. Angiographically evident thrombus is a major predictor of poorer myocardial reperfusion and this is believed to be secondary to embolization of thrombus and plaque detritus.

Based on this line of reasoning multiple thrombectomy devices have been evaluated for treating patients with STEMI. Studies evaluating these devices have been small and underpowered for clinical endpoints and have demonstrated disparate results. Further, there are major differences in thrombectomy devices and the results obtained with one class of devices may not apply to all devices. Prior meta-analyses evaluating the impact of thrombectomy devices have combined studies of thrombectomy devices with those evaluating emboli protection devices (EPDs) and have failed to establish the utility or lack thereof of thrombectomy in patients with STEMI [5-7].

The purpose of this meta-analysis was to systematically evaluate currently available data comparing thrombectomy followed by PCI with conventional PCI alone in patients with acute STEMI and to assess for differences if any between the various types of thrombectomy devices.

## Methods

We performed a computerized search to identify relevant articles from *1996 *through December *2009 *using MEDLINE (National Library of Medicine, Bethesda, Maryland), Google Scholar (Google Inc., Mountain View, California), Embase, ISI Web of Knowledge, Current Contents, International Pharmaceutical Abstracts databases, and the Cochrane Central Register of Controlled Trials. For MEDLINE we used the modified Robinson and Dickersin strategy as described by Biondi Zoccai et al [8] using the keywords "*thrombectomy", "STEMI", " thrombus aspiration", " randomized" and " PCI"*. Abstract lists from the 2005 through 2009 scientific meetings of the American Heart Association, the American College of Cardiology, the European Society of Cardiology, published review articles, editorials, and internet-based sources of information (http://www.cardiosource.com, http://www.tctmd.com, http://www.crtonline.org, http://www.theheart.org, http://www.medscape.com) were reviewed.

A study was included if it randomized patients with STEMI to aspiration thrombectomy prior to PCI or conventional PCI and provided information on 30-day outcomes. Data was independently abstracted by two reviewers (UT, IH) and disagreements were resolved by consensus. Reviewers were not blinded to study authors or outcomes. Attempt was made to retrieve the data from the original source in unpublished studies. Since no original data could be obtained in these studies, data was retrieved from earlier published meta-analysis [6]. Baseline demographic, clinical and angiographic characteristics including mean age of patients enrolled, percent of male participants, patients with diabetes mellitus, patients undergoing rescue PCI, proportion of patients with anterior wall STEMI, use of platelet glycoprotein IIb/IIIa receptor inhibitors, and mean symptom to balloon time were recorded for each study. The specific type of the thrombectomy device use in each study was recorded and devices were subcategorized based on the underlying mechanism into one of the three types (mechanical, manual, or vacuum) as previously reported [9].

We also assessed trial quality by evaluating specific elements of study design (i.e. concealment of allocation during randomization, intention to treat analysis and blinded assessment of outcome measures), but did not use a quality score given the limitations inherent to such an approach [10].

### Endpoints

Primary clinical endpoints included death, stroke, target vessel revascularization (TVR) and reinfarction. The composite endpoint point of major adverse cardiac events (MACE) at 30 days was not evaluated due to differences in definitions used in the selected studies. Myocardial perfusion was assessed by using angiographic and electrocardiographic measures. ST resolution was defined as per the study definition. Most studies defined ST resolution as more than 70% resolution in ST score [11] (defined as the sum of ST segment elevation in the leads V1 to V6, I, and aVL for anterior and II, III, aVF, and V5 to V6 for non-anterior infarctions). Single lead ST segment resolution by comparing the most prominent ST segment deviation was used in the REMEDIA trial and by Antoniucci et al [12,13]. Angiographic efficacy endpoints included post procedural rates of thrombolysis in myocardial infarction (TIMI) grade 3 flow, and myocardial blush grade (MBG).

MBG has been defined previously [14] as follows: 0, no myocardial blush or contrast density; 1, minimal myocardial blush or contrast density; 2, moderate myocardial blush or contrast density but less than that obtained during angiography of a contralateral or ipsilateral non-infarct-related coronary artery; and 3, normal myocardial blush or contrast density, comparable with that obtained during angiography of a contralateral or ipsilateral non-infarct-related coronary artery. When myocardial blush persisted ("staining"), this phenomenon suggested leakage of contrast medium into the extravascular space and is graded 0. The angiographic endpoints were independently analyzed at core labs. Of the 13 trials that recorded MBG, 9 trials [11,12,15-21] used the densitometry classification described by Van't Hof et al [14]. Two studies [22,23] utilized the dynamic method described by Gibson et al [3], while the remaining 2 studies [24,25] did not specify the exact method used. The authors had full access to the data and take responsibility for its integrity. All authors have read and agree to the manuscript as written.

### Statistical Analysis

From each trial, results were organized into a two-by-two table to permit calculation of effect sizes for aspiration thrombectomy in comparison with conventional PCI in regards to each outcome. Data on the results were collected on an "intention-to-treat" basis. When the outcome did not occur in both groups, we were unable to calculate effect sizes due to the empty cells and data were excluded from that particular trial. Continuity correction was performed when an event did not occur in one group. We used fixed-effects and random-effects models to produce across-study summary odds ratios (OR) with 95% confidence intervals. Since there was significant heterogeneity for some of the endpoints, the random-effects models are preferentially reported for the entire group although fixed effect models gave similar results. To assess the effect of individual studies on the summary estimate of effect, we did an influence analysis, in which the pooled estimates were recalculated omitting one study at a time. Subgroup analysis suggested that most of the heterogeneity could be explained by disparate results with different device classes. There was significant difference in trial size within subgroups and to avoid undue influence of small trials, and given lack of heterogeneity for most endpoints, Mantel-Haenszel fixed effect models are preferentially reported for the subgroups although random effect models gave similar results [26]. All p values were 2-tailed, with statistical significance set at 0.05. Heterogeneity was assessed by means of Cochrane Q heterogeneity test and considered significant when p value was <0.10 [27]. Publication bias was assessed by plotting a funnel plot and calculating the rank order correlation [28] and Eggers test of intercept [29]. We also calculated fail-safe N, using Rosenberg's and Orwin's method [30-32].

All analyses were performed using Comprehensive Meta-Analysis software, version 2.0 (Biostat, Englewood, New Jersey).

## Results

**Figure 1:**
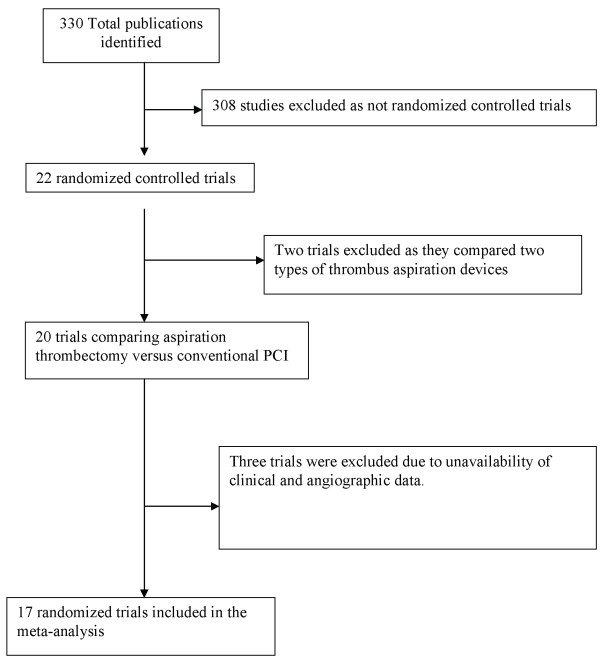
**Flow diagram depicting the selection of studies included in the meta-analysis**.

A total of 330 citations published between January 1990 and December 2009 were screened (Figure 1). Studies, which compared the two strategies without randomization [33-35] or studies prospectively enrolling consecutive patients for aspiration thrombectomy were excluded [36-38]. Two randomized trials were excluded as they compared two different types of thrombus aspiration devices [39,40]. Of the remaining 20 trials, three were excluded since they did not provide angiographic or mortality data [41-43]. Our meta-analysis thus included 17 trials that randomized patients with STEMI to thrombectomy versus conventional PCI. Of these, 14 had been published in peer-reviewed journals [11-13,15-23,44,45] while two had been published only as an abstract with information on limited endpoints [25,46]. PIHRATE trial [24] was presented at Transcatheter Cardiovascular Therapeutics meeting and the data was obtained from http://www.tctmd.com and http://www.crtonline.org. A total of 3,909 patients from 17 randomized controlled trials constituted our final study population. The characteristics of included trials and study population are shown in Table 1. Eight trials used manual aspiration thrombectomy (MAT) while mechanical and vacuum devices were used in five and four trials respectively. (Table 1). The raw clinical and angiographic events in each arm across the trials are listed in Table 2 and 3. Summary odds ratios of clinical and angiographic endpoints generated using fixed and random effect models are listed in Table 4.

**Table 1:** Baseline characteristics of studies included in the meta-analysis

	Svilaas, 2008		AIMI, 2006		DEAR-MI, 2006		Kaltoft, 2006	
	Thrombectomy	Control	Thrombectomy	Control	Thrombectomy	Control	Thrombectomy	Control
Number of patients in each arm	535	536	240	240	74	74	108	107
Male (%)	68	73	76	74	84	76	76	80
								
Age, years (mean ± SD)	63 ± 13	63 ± 13	60	60	57 ± 13	59 ± 14	65 ± 11	63 ± 13
								
Diabetes (%)	11	13	17	16	21	15	8	6
								
Glycoprotein IIb/IIIa inhibitor use (%)	93	90	95	94	100	100	96	93
								
Anterior MI* (%)	43	43	39	37	42	51	46	43
								
Mean symptom to Balloon time (minutes)	190^a^	185^a^	306^b^	300^b^	206	199	242	208
								
Thrombus (%)	49	44	65^c^	58^c^	NA	NA	69	79
								
Follow up duration, days	30	30	30	30	30	30	30	30
								
	
Device	Export		Angiojet		Pronto		Rescue	
Mechanism	Manual		Mechanical		Manual		Vacuum	
Total number of patients, (N)	1071		480		148		215	
								
Primary outcome	MBG		Infarct size		STR, MBG		Myocardial salvage	

								

	**De Luca, 2006**		**X-AMINE ST, 2005**		**REMEDIA, 2005**		**VAMPIRE, 2008**	

	Thrombectomy	Control	Thrombectomy	Control	Thrombectomy	Control	Thrombectomy	Control
Number of patients in each arm	38	38	100	101	50	49	180	175
Male (%)	71	55	76	73	90	78	80.6	77.7
								
Age, years (mean ± SD)	67 ± 14	65 ± 13	61 ± 13	62 ± 11	61 ± 13	60 ± 13	63.2 ± 11	63.5 ± 10
Diabetes (%)	24	18	25	18	22	18	23.3	29.9
Glycoprotein IIb/IIIa inhibitor use (%)	100	100	55	65	68	63	0	0
								
Anterior MI* (%)	97	100	54	50	40	51	50	52
								
Mean symptom to Balloon time (minutes)	432	456	251	264	274	300	376	426
								
Thrombus (%)	100	100	100	100	58	55	NA	NA
Follow up duration, days	180	180	180	180	30	30	240	240
								
	
Device	Diver CE		X-Sizer		Diver CE		TVAC	Export
Mechanism	Manual		Mechanical		Manual		Vacuum	Manual
Total number of patients, (N)	76		201		99		355	50
								
Primary outcome	LV remodeling		STR		MBG, STR		MBG	

	**Export, 2005**		**Dudek, 2004**		**Antoniucci, 2004**		**NON STOP, 2004**	

	Thrombectomy	Control	Thrombectomy	Control	Thrombectomy	Control	Thrombectomy	Control
Number of patients in each arm	24	26	40	32	50	50	138	131
Male (%)	NA	NA	NA	NA	82	78	79	79
Age, years (mean ± SD)	NA	NA	NA	NA	63 ± 13	66 ± 12	64 ± 12	66 ± 11
Diabetes (%)	NA	NA	NA	NA	18	16	NA	NA
								
Glycoprotein IIb/IIIa inhibitor use (%)	NA	NA	NA	NA	98	98	NA	NA
Anterior MI^¶ ^(%)	NA	NA	NA	NA	34	46	34	46
Mean symptom to Balloon time								
(minutes)	NA	NA	258	236	234	264	NA	NA
Thrombus (%)	NA	NA	100	100	NA	NA	NA	NA
Follow up duration, days	NA	NA	90	90	30	30	NA	NA

								
Device	Export		Rescue		Angiojet		Rescue	
Mechanism	Manual		Vacuum		Mechanical		Vacuum	
Total number of patients (N)	50		72		100		269	
Primary outcome	STR		TIMI, cTFC, tMPG, STR		STR		NA	

								

	**Napodano, 2003**		**Beran, 2002**		**Chevalier, 2008**		**EXPIRA, 2009**	

	Thrombectomy	Control	Thrombectomy	Control	Thrombectomy	Control	Thrombectomy	Control
Number of patients in each arm	46	46	30	31	120	129	88	87
Male (%)	83	72	73	77	80.8	81.4	64.7	55.1
Age, years (mean ± SD)	61 ± 11	64 ± 12	56 ± 10	54 ± 10	59.2 ± 13	61.2 ± 13	66.7 ± 14	64.6 ± 13
Diabetes (%)	13	13	17	13	16.7	13.2	22.7	18.4
Glycoprotein IIb/IIIa inhibitor use (%)	43	41	73	68	65.8	69.8	100	100
								
Anterior MI^¶ ^(%)	39	44	35	35	49.2	55.8	0	0
								
Mean symptom to Balloon time (minutes)	238^b^	204^b^	291	279	321.7	271.4	408	456
								
							
Thrombus (%)	100	100	100	100	NA	NA	100	100
Follow up duration, days	30	30	30	30	30	30	270	270

Device	X-Sizer		X-Sizer		Export		Export	
Mechanism	Mechanical		Mechanical		Manual		Manual	
Total number of patients, (N)	92		61		249		175	
Primary outcome	MBG		cTFC		STR, MBG		MBG, STR	
							
	**PIHRATE, 2007**							
							
	Thrombectomy	Control						
Number of patients in each arm	102	94						
Male (%)	79	81						
Age, years (mean ± SD)	61 ± 10	58 ± 10						
Diabetes (%)	12	10						
Glycoprotein IIb/IIIa inhibitor use (%)	62	63						
								
Anterior MI^¶ ^(%)	NA	NA						
								
Mean symptom to Balloon time (minutes)	195	206						
Thrombus (%)	70	70						
Follow up duration, days	180	180						
						
Device	Diver CE							
Mechanism	Manual							
Total number of patients, (N)	196							
Primary outcome	STR							

*In the studies with missing anterior MI rate, patients with left anterior descending artery as culprit artery were considered to have anterior MI; a = median; NA = Not available. b = time from symptom onset to emergency department (ED) plus time from ED to randomization; MBG = Myocardial blush grade; cTFC = Corrected TIMI frame count; tMPG = TIMI myocardial perfusion grade; STR = ST-segment resolution; TIMI = Thrombolysis in myocardial infarction grade flow; c = Grade 4 or 5

**Table 2:** Clinical events in each arm across the trials in the Meta-analysis

Study/Author	Death		Stroke		Re-infarction		Target vessel revascularization	
	Thrombectomy	Control	Thrombectomy	Control	Thrombectomy	Control	Thrombectomy	Control
Svilaas	2%	4%	0%	0%	1%	2%	5%	6%
	11	21	0	0	4	10	24	31
								
AIMI	5%	1%	1.7%	0.8%	0%	0%	2%	0.4%
	11	2	4	2	0	0	5	1
								
DEAR-MI	0%	0%	0%	0%	0%	0%	1.4%	0%
	0	0	0	0	0	0	1	0
								
Kaltoft	0%	1%	1.9%	0%	0%	1%		
	0	1	2	0	0	1	N/A	N/A
								
De Luca	0%	5%	0%	0%	3%	0%		
	0	2			1	0	N/A	N/A
								
X-AMINE	4%	4%	2%	0%	1%	3%	2%	0%
ST	4	4	2	0	1	3	2	0
								
REMEDIA	6%	6%	2.1%	2.1%	4%	4%	2.1%	2.1%
	3	3	1	1	2	2	1	1
								
VAMPIRE	1%	1%			0%	1%	0%	1%
	1	1	N/A	N/A	0	1	0	1
								
EXPORT	0%	4%						
	0	1	N/A	N/A	N/A	N/A	N/A	N/A
								
Dudek								
	N/A	N/A	N/A	N/A	N/A	N/A	N/A	N/A
								
Antoniucci	0%	0%	2%	0%	0%	0%	0%	0%
	0	0	1	0	0	0	0	0
								
NON	1%	1.5%						
STOP	2	2	N/A	N/A	N/A	N/A	N/A	N/A
								
Napodano	7%	7%	0%	0%	4%	4%	0%	0%
	3	3	0	0	2	2	0	0
								
Beran	7%	3%					0%	3%
	2	1	N/A	N/A	N/A	N/A	0	1
								
Chevalier	3%	4%	2%	0%	3%	1%	0%	0%
	4	5	2	0	3	1	0	0
								
EXPIRA	0%	1%	2%	0%	0%	0%	N/A	N/A
	0	1	2	0	0	0		
								
PIHRATE	3%	3%	N/A	N/A	0%	1%	2%	1%
	3	3			0	1	2	1

N/A = not available

**Table 3:** Angiographic events in each arm across the trials in the Meta-analysis

Study/Author	MBG 0-1		MBG 3		ST segment resolution		Post procedural TIMI 3 flow	
	Thrombectomy	Control	Thrombectomy	Control	Thrombectomy	Control	Thrombectomy	Control
Svilaas	17%	26%	46%	32%	57%	44%	86%	83%
	84	129	224	158	275	219	431	409
								
AIMI	45%	36%	27%	32%	60%	68%	91%	97%
	105	85	63	75	105	111	213	228
								
DEAR-MI	0%	7%	88%	43%	68%	50%	89%	78%
	0	5	65	32	50	37	66	58
								
Kaltoft	N/A	N/A	N/A	N/A	23%	22%	89%	88%
					22	20	93	91
								
De Luca	N/A	N/A	37%	13%	82%	55%	79%	68%
			14	5	31	21	30	26
								
X-AMINE ST	25%	25%	31%	30%	68%	53%	96%	89%
	23	23	29	28	61	50	96	90
								
REMEDIA	32%	55%	N/A	N/A	63%	37%	77%	63%
	16	27			29	18	37	31
								
VAMPIRE	17%	37%	46%	20%	N/A	N/A	88%	81%
	30	63	82	35			155	137
								
EXPORT	N/A	N/A	63%	42%	50%	12%	96%	81%
			15	11	12	3	23	21
								
Dudek	N/A	N/A	55%	38%	68%	25%	85%	86%
			22	12	27	8	34	27
								
Antoniucci	N/A	N/A	N/A	N/A	90%	72%	100%	100%
					45	36	50	50
								
NON STOP	N/A	N/A	N/A	N/A	N/A	N/A	94%	91%
							130	119
								
Napodano	15%	46%	72%	37%	83%	52%	94%	96%
	7	21	33	17	38	24	43	44
								
Beran	N/A	N/A	N/A	N/A	83%	52%	90%	84%
					19	12	27	26
								
Chevalier	28%	32%	36%	25%	74%	65%	82%	77%
	33	41	43	33	88	84	98	99
								
EXPIRA	12%	40%	70%	29%	80%	38%	N/A	N/A
	10	34	62	25	70	33		
								
PIHRATE	N/A	N/A	76%	59%	50%	41%	88%	82%
			78	55	51	39	90	77

N/A = not available

**Table 4:** Odds ratios of clinical & angiographic endpoints generated using fixed effect and random effect models

Endpoint	Summary odds ratio (random-effect model)	Summary odds ratio (fixed-effect model)	P-value for Heterogeneity
Death	0.84 (95% CI 0.54-1.29)	0.84 (95% CI 0.54-1.29)	0.66
Stroke	2.88 (95% CI 1.06-7.85)	2.88 (95% CI 1.06-7.85)	0.97
Re-infarction	0.59 (95% CI 0.29-1.22)	0.59 (95% CI 0.29-1.22)	0.80
Target vessel Revascularization	0.92 (95% CI 0.57-1.49)	0.92 (95% CI 0.57-1.49)	0.57
Myocardial blush grade 0-1	0.51 (95% CI 0.32-0.82)	0.64 (95% CI 0.54-3 0.77)	<0.001
Myocardial blush grade 3	2.42 (95% CI 1.63-3.61)	1.95 (95% CI 1.67-2.28)	<0.001
ST elevation resolution >70%	2.30 (95% CI 1.64-3.23)	1.79 (95% CI 1.53-2.08)	<0.001
Thrombolysis in myocardial infarction (TIMI) flow-3	1.41 (95% CI 1.10-1.81)	1.39 (95% CI 1.14-1.70)	0.21

### Clinical endpoints

#### 30-day mortality

Data on 30-day mortality were available in 3821 patients (98%). Mortality in the trials ranged from 0 to 7% in the thrombectomy arm and 0 to 7% in the conventional PCI arm. In the pooled estimate, 30-day death occurred in 44/1914 patients in the thrombectomy group and 50/1907 patients in the conventional PCI group.

**Figure 2:**
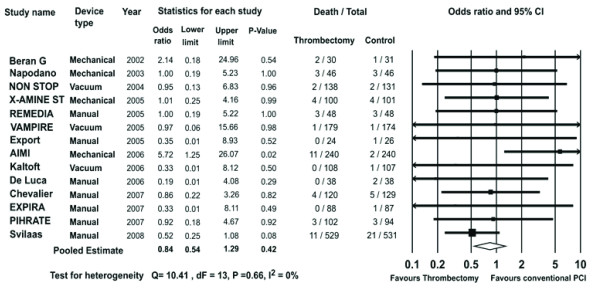
**The Forest plot of odds ratios of 30-day mortality**. Sizes of data markers are proportional to the weight of each study in the meta-analysis. Horizontal bars = 95% CI.

There was no difference in mortality between the two strategies (OR 0.84, 95% CI 0.54-1.29, P = 0.42 Figure 2).

**Figure 3:**
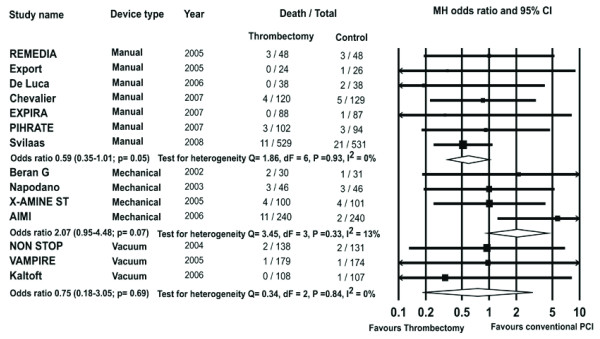
**The Forest plot of odds ratios of 30-day mortality for manual aspiration therapy (MAT), mechanical and vacuum devices**. Sizes of data markers are proportional to the weight of each study in the meta-analysis. Horizontal bars = 95% CI.

However subgroup analysis of trials studying manual, mechanical and vacuum thrombus aspiration devices showed a trend towards higher mortality with the use of mechanical devices (OR 2.07, 95% CI 0.95-4.48, P = 0.07). MAT showed significant trend towards reduction in mortality (OR 0.59, 95% CI 0.35-1.01, P = 0.05 Figure 3).

#### Stroke

Data on 30-day stroke were available in 2816 patients (72%). The incidence of stroke ranged from the 0 to 2.1% in the thrombectomy arm and 0 to 2.1% in the conventional PCI arm. Overall, stroke occurred in 14/1403 patients in the thrombectomy group and 3/1413 patients in the conventional PCI group. There was a significant increase in the likelihood of stroke with thrombectomy as compared with conventional PCI alone (OR 2.88, 95% CI 1.06-7.85, P = 0.04, P for heterogeneity = 0.97)

In subgroup analysis, there was a non-significant increased likelihood of stroke due to use of any of these devices compared to conventional PCI. (Vacuum devices OR 5.05, 95% CI 0.24-106.37, P = 0.30, Mechanical devices, OR 2.61, 95% CI 0.68-10.09, P = 0.16, and MAT, OR 2.84, 95% CI 0.51-15.65, P = 0.23).

#### Target vessel revascularization (TVR) and Reinfarction

Data on 30-day TVR were available in 3041 patients (77.8%). The incidence of TVR ranged from the 0 to 5.0% in the thrombectomy arm and 0 to 6.0% in the conventional PCI arm. Overall, TVR occurred in 35/1521 patients in the thrombectomy group and 36/1520 patients in the conventional PCI group. There was no significant difference in the likelihood of TVR with thrombectomy as compared with conventional PCI alone (OR 0.92, 95% CI 0.57-1.49, P = 0.73).

Data on 30-day reinfarction were available in 3365 patients (86.1%). The incidence of reinfarction ranged from the 0 to 4.0% in the thrombectomy arm and 0 to 4.0% in the conventional PCI arm. Overall, reinfarction occurred in 12/1684 patients in the thrombectomy group and 21/1681 patients in the conventional PCI group. There was a non-significant decrease in the likelihood of reinfarction with thrombectomy as compared with conventional PCI alone (OR 0.59, 95% CI 0.29-1.22, P = 0.16).

### Angiographic end points

#### Post procedural MBG

Data on post procedural myocardial blush were available in 13 trials. One trial reported patients with MBG 0-1, while twelve reported MBG 3 data.

#### Post procedural MBG 0-1

**Figure 4:**
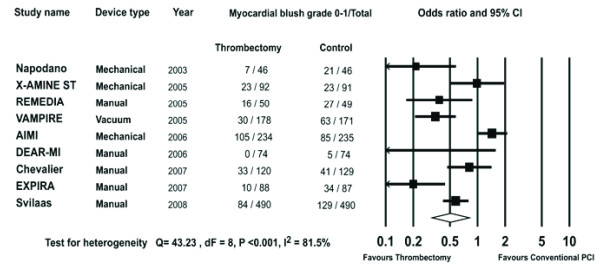
**The Forest plot of odds ratios of post procedural MBG 0-1**. Sizes of data markers are proportional to the weight of each study in the meta-analysis. Horizontal bars = 95% CI.

Data on post procedural MBG 0-1 were available in 2744 patients (70.2%). The rate of post procedural MBG 0-1 observed in the trials ranged from 0 to 45% in the thrombectomy arm and 7 to 55% in the conventional PCI arm. Overall, post procedural MBG 0-1 occurred in 308/1372 patients in the thrombectomy group and 428/1372 patients in the conventional PCI group (OR 0.51, 95% CI 0.32-0.82, P = 0.005 Figure 4).

#### Post procedural MBG 3

**Figure 5:**
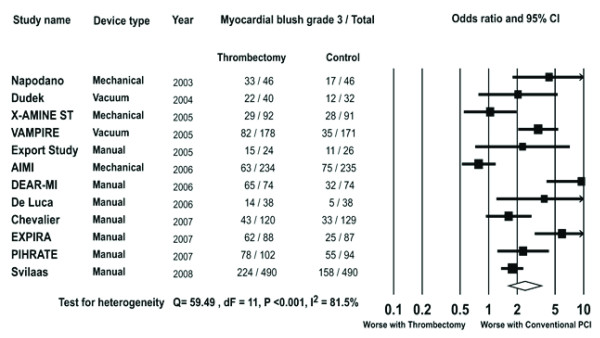
**The Forest plot of odds ratios of post procedural MBG 3**. Sizes of data markers are proportional to the weight of each study in the meta-analysis. Horizontal bars = 95% CI.

Post procedural MBG 3 was available in 3039 patients (77.7%). The rate of post procedural MBG 3 achieved in the trials ranged from 27% - 88% in the thrombectomy arm and 13% to 59% in the conventional PCI arm. Overall, post procedural MBG 3 occurred in 730/1526 patients in the thrombectomy group and 486/1513 patients in the conventional PCI group. Patients in the thrombectomy group were more likely to achieve post procedural MBG 3 compared to those undergoing conventional PCI (OR 2.42, 95% CI 1.63-3.61, P < 0.001 Figure 5).

In subgroup analysis the beneficial impact of thrombectomy on MBG 3 was seen in patients undergoing thrombectomy by vacuum device (OR 3.01, 95% CI 1.98-4.60, P < 0.001) or by MAT (OR 2.30, 95% CI 1.90-2.79, P < 0.001) but not in those treated with mechanical devices (OR 1.06, 95% CI 0.78-1.45, P = 0.70).

#### Post procedural TIMI 3 flow

**Figure 6:**
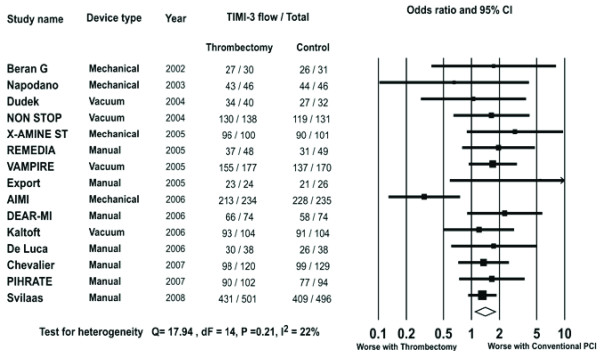
**The Forest plot of odds ratios of post procedural TIMI 3 flow**. Sizes of data markers are proportional to the weight of each study in the meta-analysis. Horizontal bars = 95% CI.

Data on post procedural TIMI 3 flow were available in 3807 patients (97.4%). The rate of post procedural TIMI 3 flow achieved in the trials ranged from 77 to 100% in the thrombectomy arm and 63 to 100% in the conventional PCI arm. Overall, post procedural TIMI 3 flow occurred in 1616/1826 patients in the thrombectomy group and 1533/1806 patients in the conventional PCI group. There was an increased likelihood of achieving post procedural TIMI 3 flow with thrombectomy compared to conventional PCI (OR 1.41, 95% CI 1.10-1.81, P = 0.007 Figure 6).

In subgroup analysis the beneficial impact of thrombectomy on post procedural TIMI 3 flow was seen in patients undergoing thrombectomy by MAT (OR 1.50, 95% CI 1.17-1.92, P = 0.001) or by vacuum device (OR 1.49, 95% CI 0.99-2.23, P = 0.05) but not in those treated with mechanical devices (OR 0.79, 95% CI 0.43-1.45, P = 0.45).

### Electrocardiographic end point

#### Post procedural ST-segment resolution>70%

Data on post procedural ST-segment resolution>70% were available in 2994 patients (76.6%). The post procedural ST-segment resolution>70% achieved in the trials ranged from 23 to 90% in the thrombectomy arm and 12 to 72% in the conventional PCI arm. Overall, post procedural ST-segment resolution>70% occurred in 923/1500 patients in the thrombectomy group and 715/1494 patients in the conventional PCI group.

**Figure 7:**
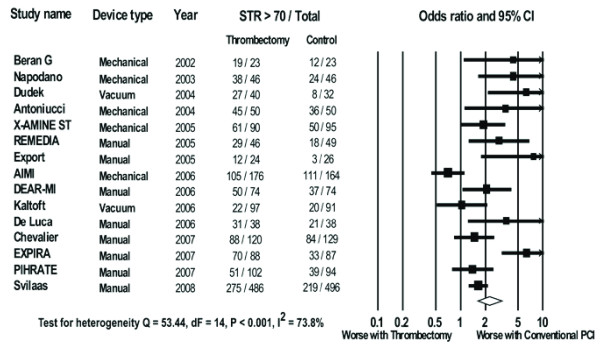
**The Forest plot of odds ratios of post procedural ST-segment resolution > 70%**. Sizes of data markers are proportional to the weight of each study in the meta analysis. Horizontal bars = 95% CI.

There was a significant increase in the likelihood of achieving post procedural ST-segment resolution>70% with thrombectomy as compared with conventional PCI (OR 2.30, 95% CI 1.64-3.23, P < 0.001, Figure 7).

In subgroup analysis the benefit was seen across all three types of thrombectomy devices (MAT OR 1.95, 95% CI 1.62-2.34, P < 0.001, vacuum devices OR 1.80, 95% CI 1.01-3.18, P = 0.05, and mechanical devices OR 1.40, 95% CI 1.02-1.91, P = 0.04).

**Figure 8:**
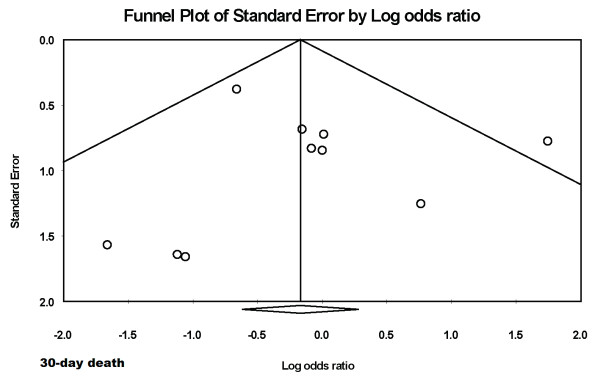
**Funnel plot for the endpoint of 30-day death**.

**Figure 9:**
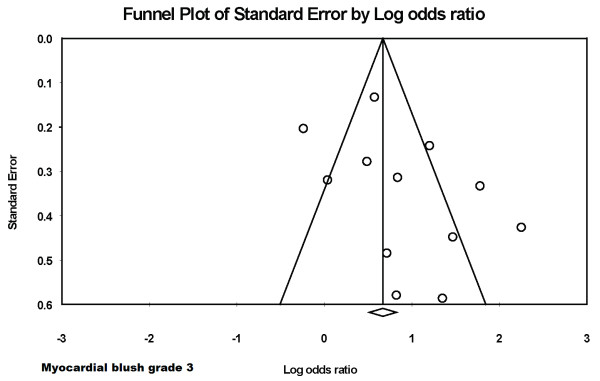
**Funnel plot for the endpoint of myocardial blush grade 3**.

There was no evidence of publication bias for the various endpoints like 30-day death (figure 8) and myocardial blush grade 3 (figure 9) on formal testing.

The calculated fail-safe N for a MBG 3 was 250. This means that 250 'null' studies would be needed in order for the combined 2-tailed p-value to exceed 0.05. Calculation of Orwin's fail-safe N, assuming a mean odds ratio of 0.75 in the missing studies was 28, suggesting that 28 studies with a mean odds ratio of 0.75 would be needed to nullify the results.

To assess the effect of individual studies on the summary estimate of effect, we performed a sensitivity analysis, in which the pooled estimates for the primary endpoint were recalculated omitting one study at a time, but this did not alter the results.

## Discussion

In this meta-analysis of seventeen randomized controlled trials, we found that compared with conventional PCI, thrombectomy devices are associated with an overall improvement in surrogate endpoints of myocardial reperfusion like STR and MBG. However, this may not be a "class effect" of thrombectomy and may vary significantly by the device used. Moreover, there appears to be a trend towards a survival benefit with the use of MAT while vacuum devices had a neutral effect and mechanical devices demonstrated a trend towards increasing mortality.

The use of thrombectomy devices however was associated with an increased risk of stroke. While it is plausible that some of the strokes are caused by embolization of aspirated thrombus and occur at time of PCI, lack of time to event data prevented us from formally assessing this hypothesis. Even with MAT, the risk of stroke even though statistically not significant, appears clinically relevant and cannot be ignored. Assuming similar base line risk, our data would suggest that treating 1000 patients with MAT would be associated with saving 14 lives (95% CI 0 to 29, P = 0.05) with a stroke occurring in 5 patients (95% CI -2 to 12, P = 0.20). These calculations are influenced by the baseline risk which in general tends to be lower in clinical trial and the absolute mortality reduction could be larger in patients with a higher baseline mortality hazard [47,48].

ST-elevation myocardial infarction is characterized by thrombus formation and occlusion of the infarct related artery (IRA). Angiographic evidence of thrombus in the IRA is associated with poor in-hospital outcomes [49,50]. Conventional PCI while attempting to open the occlusion is more likely to dislodge the thrombus downstream causing distal embolization and microvascular obstruction [51] and contribute to "no reflow phenomenon" [52]. Distal embolization has been found to occur in 15% of patients undergoing conventional PCI and is associated with reduced microvascular reperfusion of the myocardium, increased infarct size and higher long term mortality [53]. Reduced myocardial reperfusion can be tested by myocardial salvage index-defined as the proportion of jeopardized myocardium that was salvaged (measured by 99mTc-sestamibi scintigraphy), post procedural myocardial perfusion grades and persistence of ST elevation after PCI. Myocardial salvage index is an independent predictor of long-term prognosis [54] and has been shown to closely correlate with myocardial perfusion grade [55] and with resolution of ST-segment elevation on electrocardiography [56].

Myocardial blush grade (MBG) and ST resolution have emerged as valid surrogates of adequate reperfusion and poorer MBG has been consistently associated with increased infarct size, lower left ventricular function and higher long-term mortality [14,57]. Similarly, persistent ST elevation after PCI is associated with increased infarct size, decreased left ventricular function and higher mortality [58,59]. Poli et al [60] suggested that a combined analysis of MBG and STR allows a real-time grading of microvascular reperfusion of the infarct area and predicts the time-course and magnitude of LV functional recovery. They found that patients, who had neither significant MBG nor ST resolution, had poor early and late left ventricular functional recovery.

Recently Svilaas et al have shown a significant relationship between electrocardiographic and myocardial variables of reperfusion and the rate of deaths and major adverse events [20]. Therefore, thrombectomy devices were developed in an attempt to ameliorate the adverse effects of increased thrombus burden and its subsequent sequelae of decreased myocardial reperfusion. Our meta-analysis showed an overall benefit in the markers of myocardial reperfusion (MBG and STR) in thrombectomy devices consistent with earlier meta-analysis [5]. However, within the thrombectomy group MAT was more likely to be beneficial compared to mechanical devices in achieving these endpoints.

Specific features of the device and its shortcomings may play a role in explaining the differing results between manual and mechanical devices on clinical and angiographic endpoints. The significant rigidity of the mechanical catheters e.g. X-sizer, Angiojet decrease their ability to cope with tortuosity and excessive calcification proximal to the culprit lesion. Their large profile prevents crossing of tight lesions and limits its use with arteries with reference diameter >2.5 mm [9,61]. The larger profiles of these devices have the potential of inducing distal embolization and may paradoxically increase infarct size as seen in some studies [17,45].

The hydraulics of the various thrombectomy devices and the role of negative pressure developed in these devices for thrombus extraction on vessel injury is potentially important. In the Angiojet rheolytic thrombectomy system the pressurized saline jets delivered to catheter tip travel retrograde at the speed of 339 miles/hour creating a near perfect vacuum (-600 mm Hg) at the distal catheter tip for thrombus aspiration [62]. In the vacuum devices like Rescue [45] and the TVAC [35], continuous suction of 0.84 and 0.9 atmospheres respectively is applied. These high negative pressures may cause vessel collapse and injury in vessels with small caliber. Further, the mechanical devices incorporate thrombus fragmentation and aspiration and it is possible that a significant proportion of the fragmented thrombus embolizes and thus causes microvasculature injury [63]. Further, it is not clear if operator experience can explain some of the differences. The more sophisticated mechanical devices have a prolonged learning curve that is unlikely to apply to the more simpler MAT.

In comparison, the manual devices have a lower profile, are easy to operate, have short learning curve, less bulky and easy to set up by the catheterization laboratory staff and unlikely to add delay to perfusion of the occluded vessel. These differences likely explain the conflicting impact on survival seen with these devices.

The results of our meta-analysis, involving 3909 patients differ from those of the earlier work. In the meta-analysis by Deluca and colleagues (2231 patients) there was a trend towards higher mortality with thrombectomy (2.7% versus 2.1%, OR 1.28, 95% CI 0.69-2.38, P = 0.43). A similar trend was present in a meta-analysis by Burzotta et al and Kunadian et al [7] involving about 1700 and 1500 patients respectively. Our meta-analysis incorporated larger studies with more patients undergoing MAT, did not include EPDs and our findings are therefore more likely extant. On the other hand, our results corroborate the results of prior studies regarding the beneficial impact of thrombectomy devices on post procedural MBG 3 and TIMI 3 flow [6]. In a meta analysis of 9 trials Deluca et al [64] showed a significant improvement in mortality with the use of manual aspiration devices. The results of this study are similar to our study in terms of improvement in myocardial perfusion. While the directionality of the results is similar, our results did not achieve statistical significance. This may relate to differences in statistical tests use. Recently a pooled analysis of 11 trials and 2674 patients by Burzotta et al [65] based on individual patient-data, with median one year follow up showed that allocation to thrombectomy was associated with significantly lower all-cause mortality (P = 0.049). In the manual thrombectomy group, Kaplan-Meier analyses at the longest follow-up available showed that thrombectomy was associated with significantly fewer deaths (log-rank P = 0.011).

Our study results highlight the similar observations made by Bavry et al [66] regarding the increased incidence of stroke with the use of manual and mechanical aspiration devices.

The results of our meta-analysis would suggest that when thrombectomy is performed in patients undergoing PCI, the best outcome is seen with the "gentler" manual aspiration thrombectomy (MAT). Ideally, one would like to validate the results of our meta-analysis in large trials comparing manual and mechanical devices using hard clinical endpoints. Such a trial is however unlikely given the logistics and cost involved. On the other hand, it would be helpful to evaluate the survival benefits if any of manual aspiration over routine PCI in larger studies.

### Limitations

The limitations of our meta-analysis are those inherent to all meta-analyses, and they include publication bias (although tested non-significant in our study) and the difficulties in comparing the results because of the different study populations, study designs and reporting methods as well as the absence of individual patient data, which prohibits adjustment for confounding factors [67]. Data on clinical endpoints like 30-day mortality, stroke, TVR, reinfarction and angiographic endpoints like MBG 0-1, 3 were not available in some trials. Data on cardiac remodeling e.g. left ventricular ejection fraction (LVEF), left ventricular volumes and diameters were not available in most trials. Six-month data was available only in two trials and hence long-term outcomes could not be studied. Six of our studies included were not published; however, the results of sensitivity analysis did not show an impact of publication status on the study results. The event rates of clinical endpoints like death, myocardial infarction, and stroke are low in patients undergoing primary PCI as seen in large PCI registry [68]. Even with the approach of a meta-analysis the number of endpoints is low. Our meta-analysis may not be powered to detect significant differences with respect to these endpoints.

In addition, our results cannot supplant a large adequately powered randomized clinical trial.

## Conclusion

Our meta-analysis suggests a beneficial impact of thrombectomy devices on surrogate endpoints like MBG and STR. These devices have a differing impact on hard clinical endpoints with an improvement seen with MAT and worsening outcome with mechanical devices. These data suggest a need for further large clinical trials evaluating the utility of MAT in patients undergoing primary PCI.

## Competing interests

HG has been named as an inventor on patent applications filed by University of Michigan on devices that could potentially be used for thrombectomy. The remaining authors report no conflicts

## Authors' contributions

HG, UT, SC and IH were involved in conception and design of the study. HG, UT were involved in the analysis and interpretation of data. UT was involved in drafting of the manuscript. HG, MM, PMG and SC were involved in revising the study critically for important intellectual content. All the authors read and approved the final manuscript. This manuscript has not been published or submitted elsewhere.

## Pre-publication history

The pre-publication history for this paper can be accessed here:

http://www.biomedcentral.com/1471-2261/10/10/prepub
